# Panax ginseng extract rich in ginsenoside protopanaxatriol offers combinatorial effects in nitric oxide production *via* multiple signaling pathways

**DOI:** 10.1186/2193-1801-2-96

**Published:** 2013-03-09

**Authors:** Hee Yoon Ahn, So Young Hong, Ji Yeon Kim, Oran Kwon

**Affiliations:** 1 Department of Nutritional Science and Food Management, Ewha Womans University, Seoul, 120-750 Republic of Korea; 2 Department of Food Science and Technology, Seoul National University of Science and Technology, Seoul, 139-743 Republic of Korea

**Keywords:** PI3K/Akt, AMP activated protein kinase, Nitric oxide and human umbilical vein endothelial cells

## Abstract

The root of Panax ginseng *C.A. Meyer* has been shown to induce nitric oxide (NO) release resulting in a hypotensive effect. However, the main active component contributing to vascular endothelium relaxation remains uncertain. In this study, we hypothesized that multiple components of ginseng extract might have combinatory effects providing greater health benefits than a single ginsenosides. To test this hypothesis, we compared the NO-releasing and endothelial NO synthase (eNOS) activating potency of wide range of ginseng extracts (crude extract, CE; protopanaxatriol-enriched extract, TE; protopanaxadiol-enriched extract, DE) and individual ginsenosides (Rg1, Re and Rb1) in human umbilical vein endothelial cells. We found that TE had the highest potency in NO production, followed by CE, DE, and Rg1. We also observed that TE-treatment resulted in rapid activation of intracellular signaling pathways, immediate linear rise of NO, and increased eNOS activation. TE-induced activation of eNOS was abolished by pretreatment with wortmannin (inhibitor for PI3K-Akt), compound C (inhibitor for AMP activated protein kinase, AMPK) or L-NAME (inhibitor for NOS), whereas Rg1-induced eNOS phosphorylation was only partially attenuated. Further analysis revealed that TE, but not Rg1, results in AMPK phosphorylation at Thr^172^. These novel finding add evidence that the multiple components of Panax ginseng extract rich in protopanaxatriol offers combinatorial effects in NO production and vascular endothelium relaxation *via* multiple signaling pathways.

## Background

Ginseng, the root *of* Panax ginseng *C.A. Meyer*, has been widely used as both a medicine and a food in Asia for thousands of years (Helms 2004). Recently, there has been a renewed interest in investigating the health benefits of ginseng as well as its constituents by using modern techniques. Numerous studies have reported that ginseng functions as a free radical scavenger (Kang *et al*. 2006; Kitts and Hu 2000) and an immunomodulator (Lee *et al.*^2005^), contributing towards maintaining optimal health against certain chronic disease states and aging (Kitts and Hu 2000). More specifically, it has been demonstrated that ginseng had a potency to reduce blood pressure by regulating vascular tone through induction of nitric oxide (NO) release in endothelial cells (Gillis 1997). Production of NO has been known to be induced by calcium-dependent endothelial nitric oxide synthase (eNOS), whose activity is regulated under various circumstances (Hien *et al*. 2010; Edirisinghe *et al*. 2008).

To date, more than hundred of ginsenosides has been identified from Araliaceae family (Jia and Zhao 2009a, Jia et al. 2009b) and are classified into two categories based on the presence or absence of a carboxyl group at the C-6 position; protopanaxadiols (PPDs) (e.g. Rb1, Rb2, Rc, Rd ,Rg3 and Rh2) and protopanaxatriols (PPTs) (e.g. Re, Rf, Rg1, Rg2 and Rh1), respectively (Gillis, 1997). Typically, researchers have elucidated the mechanism of action of ginseng by treating human endothelial cells with highly purified individual ginsenosides. Leung *et al*. (Leung *et al.*2007a, b) found that Rg1 and Re act as functional ligands for the glucocorticoid receptor, leading to rapid NO production. Yu *et al.* (Yu *et al*. 2007) reported that Rb1 induces NO production *via* androgen receptor-mediated eNOS phosphorylation. Hien *et al*. (Hien *et al.*^2010^) investigated effects of Rg3 on endothelial NO production. Despite this large array of data for individual ginsenosides, the main active ginseng component contributing to vascular endothelium relaxation still remains uncertain.

In addition, since different ginsenosides produce differing effects, it has long been assumed that multiple components in ginseng extract can provide greater health benefits than a single ginsenoside (Kim and Kwon 2011; Low 2006). However, the combinatorial effect of multiple ginseng components in ginseng extract on NO production has not been well studied. Therefore, we investigated the study to compare ginseng extracts and individual ginsenosides for inducing NO production in human endothelial cells. To test this aim, a wide range of samples were prepared, including crude extract (CE), PPT-enriched extract (TE), PPD-enriched extract (DE) and single ginsenosides. Furthermore, to provide mechanistic explanations, we also compared the impacts of a selected extract and an equivalent amount of single ginsenoside (TE *vs* Rg1) on the activation of signaling pathways by using inhibitors.

## Results and discussion

### Comparison of NO producing ability

**Figure 1:**
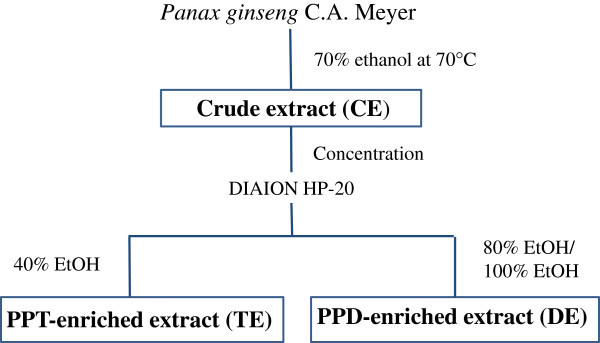
**Schematic flow diagram of the ginseng extract preparation.**

In our previous study, we demonstrated that administration of TE stimulated eNOS activation, enhanced NO production, improved vessel wall thickening, and alleviated hypertension in spontaneously hypertensive rats (Hong *et al*. 2012). In this study, as part of our continuous effort to test whether the presence of multiple active ginseng components may exert combinatorial effects, we compared the NO producing ability of a wide range of samples (Figure 1).

**Figure 2:**
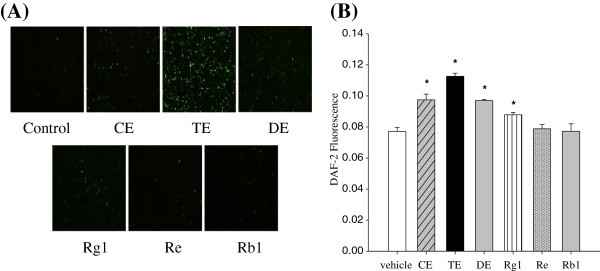
**Effect of ginseng extracts and individual ginsenosides on NO production in HUVECs.** Confluent cells were incubated with 150 μg/mL ginseng extracts (CE, TE and DE), 26 μM Rg1, 31 μM Re, or 52 μM Rb1 for 10 min. Bio-imaging of intracellular NO (**A**) and NO release (**B**) were measured using DAF-2 DA and DAF-2, respectively. Each bar represents mean ± SD from three separate wells per condition. ^*^ P < 0.05 compared with control.

First, comparison of the intracellular bio-imaging of NO was performed by using 4,5-diaminofluorescein diacetate (DAF-2DA). Human umbilical vein endothelial cells (HUVECs) were treated with each sample (150 μg/mL of CE, TE, or DE contains 25.8 μM of Rg1, 31.1 μM of Re or 52 μM of Rb1, respectively.) for 10 min, fixed, and then viewed by a fluorescence microscope. Sample concentrations were determined based on our previous *in vivo* study (Hong *et al*. 2012) and bioavailability of ginsenosides (Feng et al. 2010); Rg1 and Re concentrations were equivalent to those found in TE and Rb1 concentration was equivalent to those found in DE. Membrane permeable DAF-2 DA is taken-up by the cells and hydrolyzed by cellular esterase to form the membrane-impermeable compound 4,5-diaminofluorescein (DAF-2). As shown in Figure 2A, we observed a marked increase in intracellular DAF-2 in cells treated with TE. An increase was observed in cells treated with CE, DE and Rg1. However, little DAF-2 production was detected in cells treated with Re or Rb1.

Broillet *et al.* (Broillet *et al*. 2001) questioned whether real-time biological detection of NO concentration is really directly correlated with NO release. Therefore, to confirm our results, we measured extracellular NO release from HUVECs. Consistent with increased NO production in the cell, we detected a significant increase in DAF-2 fluorescence intensity in the extracellular media in response to TE > CE > DE > Rg1 compared to the control (Figure 2B). In contrast, Re and Rb1 treatment had no significant effect on NO release from the endothelial cells. These results support our hypothesis that multiple components in ginseng extract are more potent in inducing NO production than single ginsenosides, implicating the combinatorial interactions of these compounds. However, it should be noted that TE showed greater potency than CE and DE. This might be attributed to the lower concentration of each active ginsenoside in CE or the differential effects of PPTs on the production of NO. For individual ginsenosides, Kang *et al.* (Kang *et al*. 1995) reported that Rg1 or Re treatment induced endothelium-dependent relaxation in rat aortas, whereas Rb1 or Rc treatment did not.

**Figure 3:**
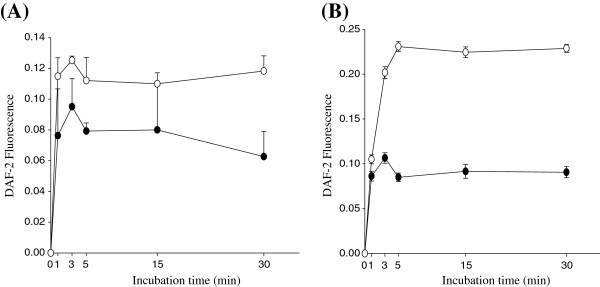
**Time-course of TE-induced NO release in HUVECs (A) and EA.hy926 (B) cells.** Cells were exposed to TE (○) or 1% DMSO (● control) and time-dependent NO release was quantified at 0, 1, 3, 5, 15, and 30 min using DAF-2. Each point represents mean ± SD from three separate wells per condition.

Ginsenosides are amphipathic in nature. Thus, they can directly interact with specific membrane proteins, triggering intracellular responses (Yue *et al*. 2007) and/or can traverse cell membranes and bind nuclear receptors primarily affecting mRNA transcription and, subsequently, protein synthesis (Attele *et al*. 1999). While transcriptional effects with subsequent modification of protein expression requires hours to days to occur (Russell *et al.*^2000^), we found that TE exposure at 150 μg/mL concentration led to an linear increase in NO production and a plateau after 5 min (Figure 3), suggesting TE -induced NO production is mediated by rapid activation of intracellular signaling pathway. It should be stressed that HUVECs (Figure 3A) and the immortalized EA.hy926 cell line (Figure 3B) showed similar patterns in NO production in response to treatment, but basal NO production is higher in EA.hy926 cells compared to primary HUVECs. Thus, EA.hy926 cells were used for the subsequent experiments.

### Comparison of PI3K/Akt-mediated eNOS phosphorylation

What intracellular signaling pathways are required for the TE-induced increase in NO production in endothelial cells? Accumulating evidence indicates that a number of protein kinases induce activation of eNOS by phosphorylating Ser^1177^ or Thr^495^ in endothelial cells. Based on previous studies (Chen *et al*. 1999), we focused on Akt- and AMP activated protein kinase (AMPK)-mediated phosphorylation of eNOS at Ser^1177^. TE and the equivalent amount of Rg1 were used for all subsequent experiments in the absence or presence of wortmannin (inhibitor of Akt signaling, 10 μM), compound C (inhibitor of AMPK signaling, 10 μM), or NG-nitro-L-arginine methyl ester (L-NAME) (inhibitor of NO synthase, 100 μM) in EA.hy926 cells.

**Figure 4:**
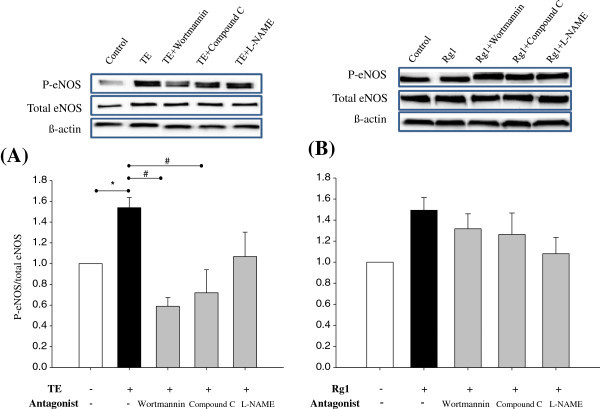
**Inhibition of TE- or Rg1-induced eNOS activation in EA.hy926 cells.** Confluent EA.hy926 cells were pretreated with wortmannin (PI3K/Akt inhibitor, 10 μM), compound C (AMPK inhibitor, 10 μM), or L-NAME (NOS inhibitor, 100 μM) for 30 min, then treated with 150 μg/mL TE (**A**) or 26 μM Rg1 (**B**) for 10 min. Representative western blots of intracellular eNOS or ß–actin are presented. Quantitative analysis of optical density of eNOS (normalized to the density of ß–actin) is shown at the bottom. Each bar represents mean ± SD, from three separate wells per condition. ^*^P < 0.05 compared with control. ^#^P < 0.05 compared with TE stimulation without inhibitor.

Consistent with increased NO release, eNOS phosphorylation was observed in cells treated with 150 μg/mL TE or 25.8 μM Rg1 for 10 min, and this effect was more pronounced in TE-treated cells (*p* = 0.01) than in Rg1-treated cells (*p* = 0.08), as shown in Figure 4. It also showed that TE-induced eNOS phosphorylation was abolished by pretreatment with inhibitors for PI3K/Akt, AMPK or NO synthase (Figure 4A). In contrast, pretreatment of these inhibitors only partially attenuated Rg1-induced eNOS phosphorylation (Figure 4B). One interpretation of these data is that the stronger signals induced by TE treatment is attributed to the activation of multiple signaling pathways (Attele *et al.*^1999^). Consistent with our results, several lines of evidence have demonstrated that Rg1 plays a role in PI3K/Akt-mediated eNOS phosphorylation leading to NO production in endothelial cells (Leung *et al*. 2006). In our previous work, we also demonstrated that TE activated eNOS phosphorylation *via* the activation of Akt in rats (Hong *et al*. 2012).

### Comparison of AMPK-mediated eNOS phosphorylation

**Figure 5:**
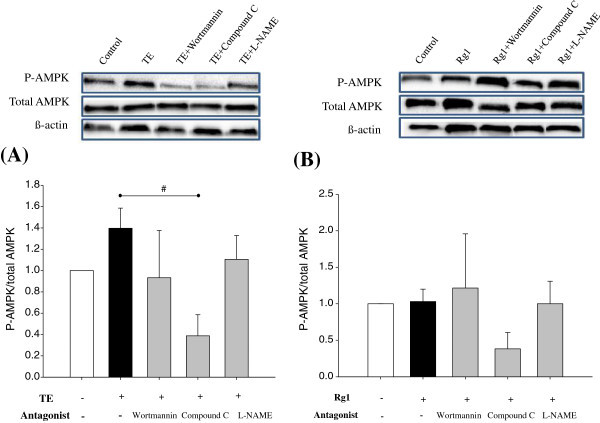
**Inhibition of TE- or Rg1-induced AMPK activation in EA.hy926 cells.** Confluent EA.hy926 cells were pretreated with wortmannin (PI3K/Akt inhibitor, 10 μM), compound C (AMPK inhibitor, 10 μM), or L-NAME (NOS inhibitor, 100 μM) for 30 min, then treated with 150 μg/mL TE (**A**) or 26 μM Rg1 (**B**) for 10 min. Representative western blots of intracellular AMPK or ß–actin are presented. Quantitative analysis of optical density of AMPK (normalized to the density of ß–actin) is shown at the bottom. Each bar represents mean ± SD, from three separate wells per condition. ^#^P < 0.05 compared with TE stimulation without inhibitor.

However, there is lack of information concerning the role of ginsenosides in relation to AMPK-mediated phosphorylation of eNOS. To dissect the signaling pathway required for phosphorylation of AMPK at Thr^172^ and subsequent phosphorylation of eNOS, we treated EA.hy926 cells with TE or Rg1 in the presence or absence of various inhibitors. Figure 5 showed that phosphorylation of AMPK markedly decreased below control level by pretreatment with compound C in both TE-treated (*p* = 0.02) and Rg1-treated (*p* = 0.08) cells, demonstrating noticeable inhibition of constitutive activation of AMPK. Interestingly, the result also revealed that there was a tendency to increase the phosphorylation of AMPK by TE treatment (p = 0.17) (Figure 5A), whereas Rg1 treatment did not affect AMPK activation (Figure 5B). As for the effect of ginsenosides on the phosphorylation of AMPK, recently, Hien *et al.* (2010) demonstrated AMPK-dependent eNOS phosphorylation in Rg3-treated endothelial cells. They also showed that Rg3-stimulated eNOS phosphorylation was reversed by AMPK inhibition. However, no report was found in the literature regarding the effect of Rg1 on AMPK-mediated eNOS phosphorylation.

## Conclusions

Our results clearly demonstrate that TE, a PPT-enriched ginseng extract, is superior in inducing NO production, compared to CE, DE, or individual ginsenosides in human endothelial cells. The stronger ability of TE to induce NO production is likely attributed to activation of multiple signal pathways, including Akt- and AMPK-mediated phosphorylation of eNOS. The novel findings of this study provide additional evidence that the diverse array of PPTs in TE likely provides better health benefits *via* combinatorial interactions to stimulate multiple signaling pathways. Importantly, the present study was conducted with the consideration of ginsenosides only, given that the NO production potency of ginseng are attributed to ginsenosides; therefore the results reported here may provide limited insight on the potency of non-ginsenoside constituents of ginseng. However, the present study may serve as a strategy to find the most appropriate preparation for plant extracts to achieve the maximum health benefits and to understand their role.

## Methods

### Reagents

Ginsenoside Re, Rb2, Rc, Rd, Rg1 and Rb1 were purchased from ChromaDex (Irvine, CA, USA). L-NAME (NO synthase inhibitor) and compound C (AMPK inhibitor) were purchased from Cayman Chemical (Ann Arbor, MI, USA). Wortmannin (PI3K-Akt inhibitor) was purchased from Sigma (St. Louis, MO, USA). Antibodies (eNOS, phospho-eNOS^Ser 1177;^ AMPK, and AMPK^Thr172^) were purchased from Cell Signaling Technology (Beverly, MA, USA). HUVECs, the immortalized HUVEC cell line EA.hy 926 and culture medium were purchased from the American Type Culture Collection (Bethesda, MD, USA). DAF-2 and DAF-2 DA were purchased from Alexis Biochemicals (Grünberg, Germany) and Cayman Chemical, respectively.

### Preparation of ginseng extracts

The CE, TE and DE were kindly provided by CJ Cheiljedang Corp. (Seoul, Korea). Briefly, dried ginseng (Panax ginseng *C.A. Meyer*) roots were cut into small pieces and refluxed in 70% ethanol. After removing ethanol, CEs were eluted on DIAION HP-20 ion exchange resin (Mitsubishi Chemical Co., Tokyo, Japan) to obtain TE and DE by 40% ethanol elution and by both 100% and 80% ethanol elution, respectively (Figure 1). The yields were found to be 50%, 1% and 5% for CE, TE and DE, respectively. The major ginsenosides in freeze-dried extracts were quantified by comparison with standards using an Agilent 1100 HPLC system (Palo Alto, CA, USA) equipped with a reversed-phase column (Venusil XBP C18, 250 X 4.6 mm, i.d. 5 μm, Agela Technology, Newark, DE, USA) (Table 1).

**Table 1:** **Content of major ginsenosides in test materials**

Preparations	Protopanaxatriol type		Protopanaxadiol type			
	Rg1 (mg/g)	Re (mg/g)	Rb1 (mg/g)	Rb2 (mg/g)	Rc (mg/g)	Rd (mg/g)
Crude extract (CE)	5.9	18.2	25	13.5	27.3	9.6
PPT-enriched extract (TE)	136.7	325.6	63.1	29.2	73.2	11.5
PPD-enriched extract (DE)	14.1	26.9	229.9	132.2	261.2	81.3

### Cell culture and treatments

For NO production assay, confluent cells in 12-well plates were serum-starved overnight and treated with the respective samples in Ca^+2^-containing phosphate buffered saline for 10 min at 37°C. For inhibitor assays, confluent cells in 100 mm dishes were serum-starved overnight, pretreated with different inhibitors (L-NAME, 100 μM; wortmannin, 10 μM; compound C, 10 μM) for 30 min, and then treated with TE or Rg1 for 10 min. Ginseng extracts and ginsenosides were prepared fresh by diluting a 100-fold concentrated stock solution prepared in dimethyl sulfoxide.

### Measurement of intracellular and extracellular NO production

For intracellular NO production, confluent cells were pre-incubated with 5 μM DAF-2 DA for 30 min at 37°C in darkness, rinsed with fresh suspension buffer to remove excess fluorophore, and treated with the respective samples for 10 min. The cells were fixed in 2% paraformaldehyde and green fluorescence zimages obtained using a fluorescent microscope (Nikon ECLIPSE TS 100, Nikon, Tokyo, Japan) at 495 nm excitation and 515 nm emission wavelength (Kojima *et al.*^1998^). For extracellular NO release, DAF-2 (1 μM) was added in assay medium for 5 min at 37°C in darkness after treatment with respective samples. Aliquots of the solutions were sampled and fluorescence was measured using a Thermo Scientific Fluorometer (Barrington, IL, USA) at 495 nm excitation and 515 nm emission wavelength (Leikert *et al.*^2001^).

### Western blot analysis

Cells were stimulated with respective samples for 10 min and then lysed in lysis buffer. Equal quantities of protein were resolved by SDS-polyacrylamide gel electrophoresis and transferred onto polyvinylidene difluoride membranes (Bio-Rad, Hercules, CA, USA). The proteins were probed with the indicated primary antibodies, and then incubated either goat anti-rabbit or goat anti-mouse secondary antibody. Bands were visualized using the West-one Western Blot Detection System (iNtRON Biotechnology, Korea). Band intensity was quantified using ChemiDoc XRS + Systems with Image Lab software (Bio-Rad, Hercules, CA, USA) and normalized to β-actin (Santa Cruz Biotechnology) densitometric values.

### Statistical analysis

All data shown are representative of at least three experiments that yielded similar results. Data are presented as the mean of triplicate samples with error bars indicative of the standard deviations. The numerical results were analyzed using one-way analysis of variance with *post hoc* Dunnett’s multiple range tests. *P <*0.05 was considered statistically significant. Statistical analyses were performed using the SAS package version 9.2 (SAS Institute, Cary, NY).

## Abbreviations

AMPK: AMP activated protein kinase
CE: Crude extract
DAF-2: 4,5-diaminofluorescein
DAF-2DA: DAF-2 diacetate
DE: PPD-enriched extract
eNOS: Endothelial nitric oxide synthase
HUVECs: Human umbilical vein endothelial cells
L-NAME: NG-nitro-L-arginine methyl ester
NO: Nitric oxide
PPD: Protopanaxadiol
PPT: Protopanaxatriol
TE: PPT-enriched extract.
